# Retinal hemodynamic and caliber biomarkers for the assessment of cerebral small vessel disease burden: insights from fundus fluorescein angiography

**DOI:** 10.1186/s12883-026-05006-0

**Published:** 2026-05-25

**Authors:** Lingxu Xu, Xiangwei Xiong, Yue Zhou, Xiao Zhao, Yutong Wu, Cheng Ye, Zhaoyou Meng

**Affiliations:** 1 Department of Neurology, Second Affiliated Hospital of Army Medical University, Chong Qing, China; 2 Department of Ophthalmology, Second Affiliated Hospital of Army Medical University, Chong Qing, China; 3 Department of Neurology, The 922th Hospital, Joint Logistic Support Force, Hengyang, Hunan 421002 China

**Keywords:** Cerebral small vessel disease, Fundus fluorescein angiography, Retinal microvasculature, CRAE, CRVE

## Abstract

**Background:**

Retinal microvasculature serves as a promising window to assess cerebral small vessel disease (CSVD). However, the association between comprehensive fundus fluorescein angiography (FFA) biomarkers and the total CSVD burden score remains to be fully elucidated.

**Aimes:**

To investigate the relationship between the total CSVD burden score and FFA-derived retinal biomarkers, and to identify independent retinal indicators for evaluating CSVD severity.

**Methods:**

This retrospective study enrolled 120 patients. The CSVD burden score was evaluated based on lacunes of presumed vascular origin, white matter hyperintensities, enlarged perivascular spaces, and cerebral microbleeds. FFA parameters included dynamic hemodynamic indicators (arm-retinal circulation time [ARCT], retinal circulation time [V1-ARCT], retinal venous transit time [V2-V1]) and static vascular caliber indicators (central retinal artery equivalent [CRAE], central retinal vein equivalent [CRVE], arteriolar-to-venular ratio [AVR]), as well as arteriovenous nicking and vascular leakage. Correlation and multivariable regression analyses were performed.

**Results:**

Across CSVD burden groups, significant differences were detected in age, hypertension, hyperlipidemia, key neuroimaging markers, ARCT, V1-ARCT, CRAE, CRVE, and AVR, whereas gender, diabetes, arteriovenous nicking, vascular leakage, and V2-V1 showed no significant differences. Spearman analysis showed that the CSVD burden score was negatively correlated with CRAE and AVR, and positively correlated with CRVE and V1-ARCT. Multivariable analysis identified CRAE, CRVE, and V1-ARCT as independent factors associated with the CSVD burden score: lower CRAE, higher CRVE, and prolonged V1-ARCT were related to higher CSVD burden.

**Conclusion:**

Static vascular caliber (CRAE, CRVE) and dynamic retinal circulation time (V1-ARCT) are independently associated with CSVD burden. The combined use of these FFA biomarkers provides a promising tool for the early assessment and noninvasive monitoring of CSVD, supporting further clinical translation.

## Introduction

Cerebral small vessel disease (CSVD) is a clinical syndrome caused by pathological changes in the small vessels supplying the brain parenchyma, manifesting as lacunar stroke, motor dysfunction, and cognitive impairment [1]. CSVD is highly prevalent in the elderly and confers a heavy societal burden [2]. Importantly, CSVD is often clinically silent in early stages, and its diagnosis mainly relies on late-stage MRI markers, including recent small subcortical infarcts(RSSI), lacunes of presumed vascular origin (LPVO), white matter hyperintensities (WMH), enlarged perivascular spaces(EPVS), and cerebral microbleeds(CMB) [3–4]. However, these structural MRI abnormalities appear only after substantial vascular injury has occurred, limiting early screening in high-risk populations [5]. Therefore, identifying reliable, noninvasive biomarkers for the early detection and monitoring of CSVD is urgently needed.

The retinal and cerebral small vessels share similarities in embryological origin, anatomical structure, and physiological function, as a result, the retinal microvasculature has emerged in recent years as a window to observe cerebrovascular health [6–8]. Recent large-scale neuroimaging studies have demonstrated widespread and disorder-specific alterations in cortical thickness and surface area, which reflect important structural adaptations of the brain in response to microvascular damage and dysfunction [9–10]. The Rotterdam Study demonstrated a significant positive association between wider retinal venular diameter and WMH [11]. In CSVD patients with diabetes, the arteriolar to venular ratio (AVR) was found to be significantly suggestive of CSVD, independent of other risk factors [12]. Furthermore, parameters such as retinal nerve fiber layer thickness and retinal vascular density have also been significantly associated with CSVD [13–16].

Fundus fluorescein angiography (FFA) is a powerful tool that enables dynamic visualization of retinal perfusion and simultaneous assessment of both vascular caliber and microcirculatory function [17]. To date, the combined value of FFA-derived static caliber and dynamic hemodynamic parameters for evaluating CSVD burden remains unclear. This retrospective study aimed to explore the association between FFA-based retinal biomarkers (including both static vascular caliber and dynamic circulation parameters) and MRI-defined CSVD burden score, and to identify independent retinal indicators for CSVD severity. Our findings may support the use of retinal microvascular assessment as a noninvasive strategy for the early detection of CSVD.

## Methods

### Study population

A total of 120 patients with CSVD were enrolled in this single-center retrospective study, utilizing data from the Department of Neurology, The Second Affiliated Hospital of Army Medical University for the period from January 2018 to November 2024. The inclusion criteria were: (1) Aged≥40years; (2) Conformed to the imaging criteria for CSVD, defined as the presence of at least one marker on MRI, according to the standards set forth in the *2021 ESO Guideline on Covert Cerebral Small Vessel Disease* [18] or the STRIVE-2 consensus [3]; (3)Arteriolosclerotic Type. The exclusion criteria were: (1) Loss of medical records, incomplete neuroimaging data, poor quality FFA images (including motion artifacts, defocus, media opacity, or insufficient vascular filling), an interval exceeding 2 months between FFA and MRI examinations. (2) Patients with genetic CSVD (e.g.CADASIL), cerebral amyloid angiopathy, migraine-related isolated white matter hyperintensities, or other secondary causes of CSVD.(3)Severe stenosis (≥ 70%) of the internal carotid artery distal to the ophthalmic segment, intracranial tumors, intracranial hypertension, arteriovenous malformations, large area cerebral infarction and severe dysfunction of major organs.(4)A history of intraocular surgery or any of the following ophthalmic conditions: glaucoma, branch retinal artery or vein occlusion, diabetic macular edema, diabetic retinopathy, optic neuritis, ischemic optic neuropathy, pupillary seclusion, central serous chorioretinopathy, or other diseases known to significantly affect retinal vascular caliber or morphology.

### Data collection

Clinical data were obtained from the hospital’s electronic medical record system, comprising sex, age, smoking, hypertension, diabetes, and hyperlipidemia. Raw MRI data of the brain, including T1WI, T2WI, fFLAIR, DWI and SWI, were acquired from the Picture Archiving and Communication System. Original FFA images were retrieved from the Department of Ophthalmology’s German Heidelberg Spectralis system.

### MRI data processing

**Fig. 1:**
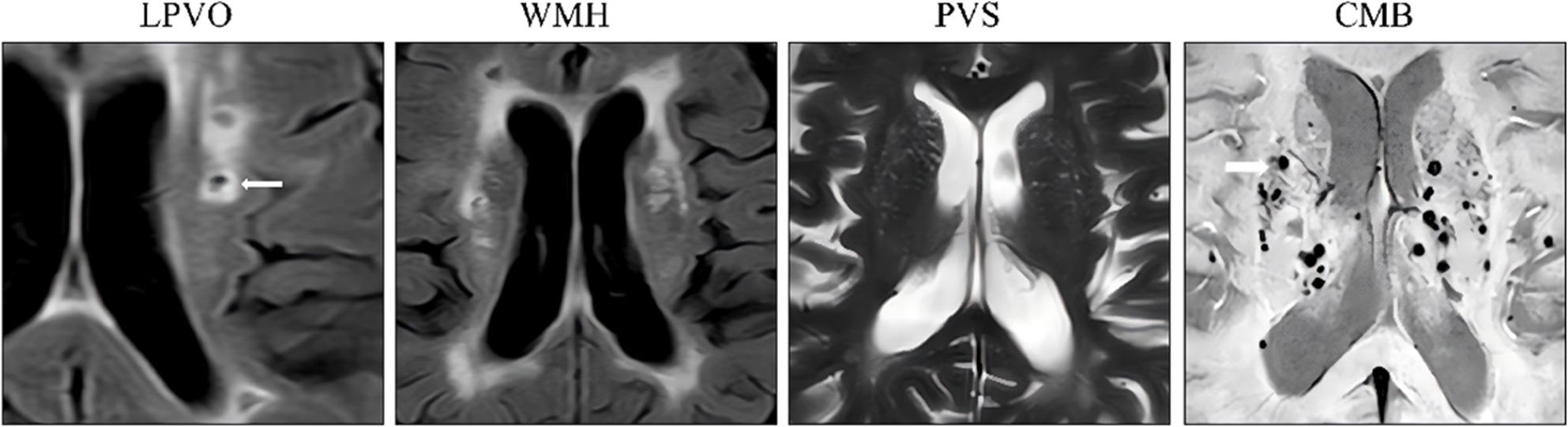
Representative MRI findings of CSVD lesions

Brain MRI images were independently and blindly assessed by two experienced neurologists who were blinded to all clinical information. According to the STRIVE-2 criteria, they identified LPVO, WMH, EPVS, and CMB. The severity of each imaging manifestation was rated using corresponding standardized scales. Any discrepancies in the grading of LPVO, WMH, EPVS, and CMB were resolved by discussion with a senior third reviewer to reach a consensus. All four types of imaging findings are illustrated in Fig. 1.

All images were selected from the present study cohort.

LPVO typically represent the residual imaging manifestations following various acute cerebral insults, such as RSSI, CMI or incidental DWI positive lesions. These parenchymal defects are characterized as round or ovoid cavities measuring 3–15 mm in diameter, predominantly located in the basal ganglia or centrum semiovale regions [19]. The following parameters of LPVO were systematically documented: count, spatial location, size, and morphological characteristics.

WMH manifest as punctate or patchy areas of increased signal intensity on T2WI and FLAIR sequences [20].These lesions were systematically evaluated using the established Fazekas visual rating scale, which categorizes them into two distinct anatomical distributions: periventricular white matter hyperintensities (PVWMH) and deep white matter hyperintensities (DWMH). For PVWMH, grades correspond to: 0 (none), 1 (caps/pencil-thin linings), 2 (smooth halos), and 3 (irregular extensions into deep white matter). For DWMH, grades indicate: 0 (none), 1 (punctate foci), 2 (beginning confluence), and 3 (large confluent areas) [21].

The severity of EPVS was evaluated using the visual semi-quantitative rating scale established by Potter [22]. For BG-EPVS and CS-EPVS, grades correspond to: 0 (none), 1 (1ཞ10 EPVS), 2 (11ཞ20 EPVS), 3 (21ཞ40 EPVS) and 4(>40 EPVS). For midbrain region, grades correspond to:0(absence of detectable EPVS),1(definite presence of EPVS) [23].

The assessment of CMB was performed using a manual counting method. This approach quantified the total number of CMB and precisely mapped their anatomical locations.

CSVD Burden Score: based on the above four MRI manifestations, the CSVD Burden Score ranging from 0 to 4 points. 1 point was assigned for LPVO if ≥ 1 lacune was present. 1 point was allocated for CMB if ≥ 1 microbleed was detected. 1 point was attributed to WMH if either PVWMH reached Fazekas grade 3, or DWMH reached Fazekas grade ≥ 2. 1 point was designated for enlarged perivascular spaces in BG-EPVS if the grade was ≥ 2 [24].

### FFA data processing

FFA images were independently and blindly analyzed by two experienced ophthalmologists who were blinded to clinical data. Discrepancies in measuring hemodynamic parameters, counting arteriovenous nicking sites, and identifying vascular leakage were resolved through consultation and discussion between the two ophthalmologists.

Hemodynamic Parameters: The following parameters were recorded, the arm-retinal circulation time (ARCT), defined as the duration until the initial appearance of contrast in the retinal arteries. Record the time to complete laminar flow filling in venous branches (V1) and the time to complete venous branch filling (V2). Based on these measurements, calculate the Retinal circulation time (V1-ARCT) and the retinal venous transit time (V2-V1).

The number of arteriovenous nicking sites in both eyes and the presence of vascular leakage were recorded manually.

**Fig. 2:**
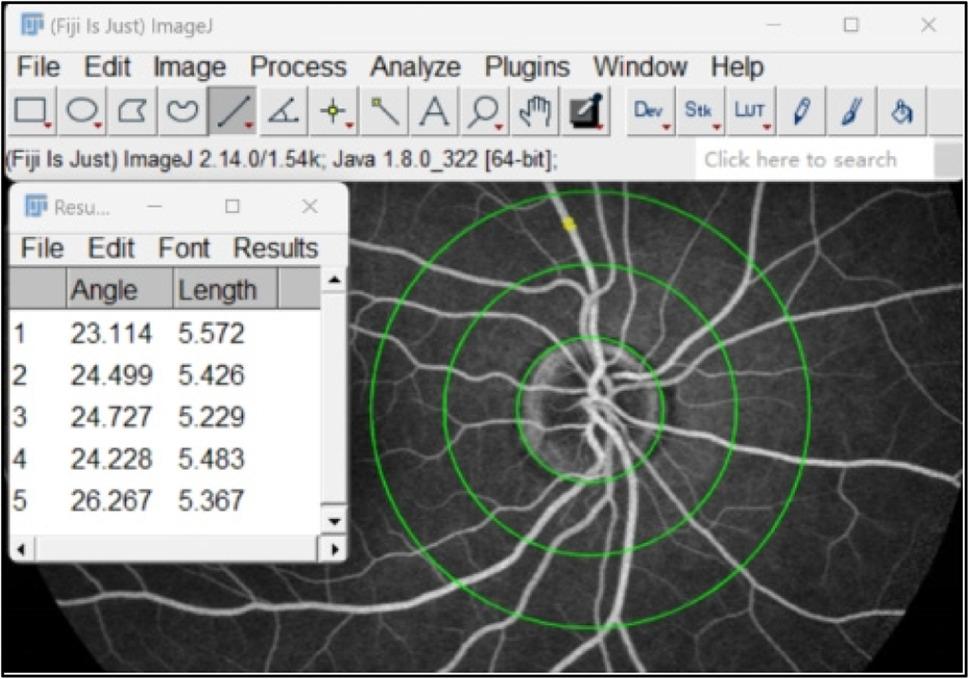
Schematic diagram of the measurement protocol on FFA images

Central retinal artery equivalent (CRAE), Central retinal vein equivalent (CRVE), and the Arteriolar to venular ratio (AVR): using Fiji software (National Institutes of Health, USA) to measure the CRAE and CRVE [25]. FFA images with sufficient arterial-venous filling, clear optic disc, and well-visualized macula were included. The six largest arteries and veins were selected within a circular zone ranging from 0.5 to 1.0 disc diameter from the optic disc margin. Images were magnified 6-fold; measurements were avoided at vascular bifurcations and arteriovenous crossings and each vessel was measured five times (Fig. 2). To ensure reliability, vessel diameters were independently assessed by two trained physicians. Inter-rater reliability was evaluated by intraclass correlation coefficient (ICC) and showed good consistency (ICC = 0.88). Intra-rater reliability was tested in 10% of randomly selected images (ICC = 0.92). The average of the two measurements was used as the final diameter.The measured diameters were sorted in ascending order and designated as W1, W2, . W5, W6. Vessels were paired sequentially as (W1, W6), (W2, W5) and (W3, W4), then calculate The CRAE and CRVE using the modified Parr-Hubbard formula [26].

$$\begin{array}{c}\mathrm{CRAE}=\;0.88\ast\left(\mathrm W_{\mathrm i}^2+\mathrm W_{\mathrm j}^2\right)^{1/2}\\{\mathrm{CRVE}=\;0.95\ast\left(\mathrm W_{\mathrm i}^2+\mathrm W_{\mathrm j}^2\right)^{1/2}}\\{\mathrm{AVR}=\mathrm{CRAE}/\mathrm{CRVE}}\end{array}$$

The calculation was iterated twice to obtain the CRAE, CRVE and AVR, the final result was taken as the mean value from both eyes.

### Statistical analysis

All statistical analyses were performed using R software (version 4.5.2). Continuous variables were assessed for normality using the Kolmogorov-Smirnov test. Normally distributed data are presented as mean ± standard deviation (x ± s) and were compared using the Student’s t-test or ANOVA. Categorical data are expressed as numbers (percentages), using the chi-square (χ²) test or Fisher’s exact probability test. Non-normally distributed continuous variables and ordinal data are presented as median [M (P25, P75)] and were analyzed using the Mann-Whitney U test. Multivariable analysis was performed using a multivariable ordinal logistic regression model. Correlation analysis was conducted using Spearman’s rank correlation analysis. Correction for multiple comparisons was not performed to avoid overcorrection and loss of statistical power. A two-sided P-value ≤ 0.05 was considered statistically significant.

## Results

### Clinical profile stratified by CSVD burden score

The 120 patients with CSVD were stratified into five groups based on Burden Score: score 0 (*n* = 37), score 1 (*n* = 20), score 2 (*n* = 18), score 3 (*n* = 23), and score 4 (*n* = 22). Comparisons among the CSVD Burden Score groups revealed no statistically significant differences in gender, diabetes, arteriovenous nicking sites, vascular leakage and the retinal venous transit time (V2-V1)(*P* > 0.05). However, significant differences were observed in age, hypertension, hyperlipidemia, number of lacunes, visibility of EPVS in the midbrain, PVWMH, DWMH, BG-EPVS, CS-EPVS, CMB, ARCT, V1-ARCT, CRAE, CRVE, and AVR (*P* < 0.05). Detailed results are presented in Table 1.

**Table 1:** Clinical profile stratified by CSVD burden score

	0 score (*n* = 37)	1 score (*n* = 20)	2 score (*n* = 18)	3 score (*n* = 23)	4 score (*n* = 22)	*P* value
Man, *n*(%)	23(62.16)	12(60.00)	12(66.67)	17(73.91)	19(86.36)	0.289
Age, years	48.16 ± 19.12	58.75 ± 14.38	61.33 ± 9.94	63.70 ± 10.52	65.64 ± 9.66	**<0.001**
Hypertension, n(%)	14(37.84)	8(40.00)	14(77.78)	14(60.87)	17(77.27)	**0.005**
Diabetes, n(%)	7(18.92)	1(5.00)	4(22.22)	2(8.70)	4(18.18)	0.464
Hyperlipidemia, n(%)	4(10.81)	2(10.00)	7(38.89)	10(43.48)	3(13.64)	**0.009**
LVPO, n	0 ± 0	0.05 ± 0.22	0.11 ± 0.32	0.57 ± 0.84	2.05 ± 1.46	**<0.001**
PVWMH, grade	1[0–1]	1.5 [1–2]	3 [2–3]	3[3–3]	3 [3–3]	**<0.001**
DWMH, grade	0 [0–1]	1 [0–1]	2 [1–3]	3 [2–3]	3 [2–3]	**<0.001**
BG-EPVS, grade	1 [1–1]	1.5 [1–2]	2 [1–2]	2 [2–3]	3.5 [2–4]	**<0.001**
CS-EPVS, grade	1 [1–2]	2 [1–3]	2 [2–3]	2 [1–3]	3 [1–4]	**0.002**
EPVS in the midbrain, n(%)	7(18.92)	1(5.00)	5(27.78)	11(47.83)	14(63.64)	**<0.001**
CMB, n(%)	0(0.00)	4(20.00)	9(50.00)	21(91.30)	22(100.00)	**<0.001**
arteriovenous nicking sites, n	1.92 ± 1.75	2.20 ± 2.19	1.89 ± 1.94	1.61 ± 1.95	2.09 ± 1.93	0.878
ARCT, s	14.49 ± 3.26	14.91 ± 4.04	16.16 ± 3.86	16.59 ± 5.30	17.65 ± 3.46	**0.033**
V1-ARCT, s	1.62 ± 0.60	1.71 ± 0.64	1.83 ± 0.50	1.80 ± 0.81	2.82 ± 0.90	**<0.001**
V2-V1, s	11.47 ± 3.24	10.17 ± 3.56	12.93 ± 3.78	12.81 ± 4.63	12.06 ± 3.30	0.110
vascular leakage, n(%)	0(0.00)	0(0.00)	1(5.56)	0(0.00)	2(9.09)	0.083
CRAE, µm	116.37 ± 11.34	114.75 ± 14.26	112.15 ± 16.26	109.55 ± 17.89	105.39 ± 10.72	**0.046**
CRVE, µm	191.30 ± 15.96	192.88 ± 17.33	204.37 ± 22.72	215.06 ± 24.63	234.27 ± 15.01	**<0.001**
AVR, ratio	0.61 ± 0.05	0.60 ± 0.08	0.55 ± 0.06	0.51 ± 0.07	0.45 ± 0.04	**<0.001**

Continuous data are expressed as mean ± standard deviation, categorical data as number (%), and ordinal data as median (Q1, Q3)

The bolded indicates statistically significant at *p* < 0.05

*LVPO *Lacunes of presumed vascular origin, *PVWMH *periventricular white matter hyperintensities, *DWMH *deep white matter hyperintensities, *BG-EPVS *enlarged perivascular space in basal ganglia, *CS-EPVS *enlarged perivascular space in centrum semiovale, *CMB *Cerebral microbleeds, *ARCT *Arm retinal circulation time, *V1-ARCT *retinal circulation time, *V2-V1 *retinal venous transit time, *CRAE *central retinal artery equivalent, *CRVE *central retinal vein equivalent, *AVR *arteriolar to venular ratio

### Correlations of CSVD burden score and retinal biomarkers

To investigate the relationship between retinal microvascular parameters obtained from FFA) and the CSVD burden score, we performed a correlation analysis. Spearman correlation analysis revealed that the CSVD Burden score was negatively associated with CRAE and AVR, and positively associated with ARCT, V1-ARCT, and CRVE (Table 2). After adjusting for potential confounders including gender, age, hypertension, diabetes and hyperlipidemia, the CSVD Burden score remain negatively associated with CRAE, AVR, and positively associated with V1-ARCT and CRVE (Table 3).

**Table 2:** Correlation analysis of CSVD burden score and retinal biomarkers

Retinal biomarkers	*r*	*P* value
V2-V1	0.124	0.175
CRAE	-0.291	**0.001**
ARCT	0.331	**<0.001**
V1-ARCT	0.394	**<0.001**
CRVE	0.665	**<0.001**
AVR	-0.692	**<0.001**

The bolded indicates statistically significant at *p* < 0.05

*V2-V1 *retinal venous transit time, *CRAE *central retinal artery equivalent, *ARCT *Arm retinal circulation time, *V1-ARCT *retinal circulation time, *CRVE *central retinal vein equivalent, *AVR *arteriolar to venular ratio

**Table 3:** Adjusted correlation analysis of CSVD burden and retinal biomarkers

Retinal biomarkers	Partial Correlation Coefficient	*P* value
V2-V1	0.067	0.479
CRAE	-0.204	**0.029**
ARCT	0.134	0.154
V1-ARCT	0.343	**<0.001**
CRVE	0.593	**<0.001**
AVR	-0.635	**<0.001**

The bolded indicates statistically significant at *p* < 0.05

*V2-V1 *retinal venous transit time, *CRAE *central retinal artery equivalent, *ARCT *Arm retinal circulation time, *V1-ARCT *retinal circulation time, *CRVE *central retinal vein equivalent, *AVR *arteriolar to venular ratio

### Multivariable logistic regression analysis of CSVD burden score

To further identify key biomarkers associated with CSVD, we employed a multivariable logistic regression model. During the modeling process, significant multicollinearity (VIF > 10) was detected between the AVR and its components, CRAE and CRVE. To mitigate potential estimation bias, the AVR was excluded from the final model, while CRAE and CRVE parameters that directly reflect retinal vascular caliber were retained.

The results identified CRAE, CRVE, age and V1-ARCT as significant correlates of CSVD. Specifically, CRAE demonstrated a negative correlation with the CSVD burden score (β = -0.076, OR = 0.927, 95% CI: 0.898–0.958, *P* < 0.001). In contrast, CRVE (β = 0.082, OR = 1.086, 95% CI: 1.059–1.112, *P* < 0.001) and V1-ARCT (β = 0.677, OR = 1.968, 95% CI: 1.093–3.543, *P* = 0.024) showed positive correlations with the CSVD Burden Score (Table 4).

**Table 4:** Multivariable logistic regression analysis of CSVD burden score

Biomarkers	β	Standard Error	OR	95% CI	*P* value
Gender	0.025	0.439	1.025	[0.433,2.425]	0.955
Age	0.050	0.015	1.052	[1.021,1.083]	**<0.001**
Diabetes	-0.632	0.550	0.531	[0.181,1.563]	0.251
Hypertension	0.194	0.409	1.214	[0.545,2.703]	0.636
Hyperlipidemia	-0.067	0.453	0.935	[0.385,2.271]	0.882
ARCT	-0.029	0.050	0.972	[0.881,1.071]	0.563
V1-ARCT	0.677	0.300	1.968	[1.093,3.543]	**0.024**
V2-V1	-0.004	0.050	0.996	[0.903,1.100]	0.944
CRVE	0.082	0.012	1.086	[1.059,1.112]	**<0.001**
CRAE	-0.076	0.016	0.927	[0.898,0.958]	**<0.001**

The bolded indicates statistically significant at *p* < 0.05

*ARCT *Arm retinal circulation time, *V1-ARCT *retinal circulation time, *V2-V1 *retinal venous transit time, *CRVE *central retinal vein equivalent, *CRAE *central retinal artery equivalent

## Discussion

This study investigated the relationship between retinal microvascular parameters derived from FFA and CSVD burden score based on MRI image. Our principal findings indicate that specific retinal biomarkers: CRAE, CRVE, and V1-ARCT, exhibit significant correlations with CSVD severity, even after adjusting for major vascular risk factors. These results reinforce the potential role of retinal microvasculature as a window into cerebral small vessel health and provide new insights into early screening strategies for CSVD.

The retinal microvascular are non-invasive markers of brain health has been widely studied [27–31]. The 3 C Dijon study showed that geometric leftacteristics of the retinal microvasculature are associated with increased burden of MRI-CSVD and possibly worse executive function(smaller arteriolar caliber with larger WMH, greater venular tortuosity with lacunes) [32]. In addition, diabetes patients with moderate to severe burden of CSVD were more likely than those with mild burden of CSVD to have narrowed CRAE, widened CRVE, and lower AVR [33]. Similarly, the ARIC Study provides evidence that midlife focal arteriolar narrowing of the retina is associated with late life CSVD markers of brain changes [34]. In our study, The significant associations observed between the CSVD burden score and traditional vascular risk factors, older age, hypertension and hyperlipidemia, align with their well established roles in the pathogenesis of CSVD. Notably, we found no significant associations of CSVD burden with sex, diabetes, arteriovenous nicking, vascular leakage, or V2-V1. The lack of a significant difference in the prevalence of diabetes across CSVD score groups in our study can be attributed to the stringent exclusion criteria, which specifically omitted patients with diabetic retinopathy to avoid its confounding effect on retinal vascular caliber, resulting in a smaller subset of diabetic patients eligible for inclusion. Similarly, the absence of a significant association for vascular leakage(a direct indicator of blood retinal barrier disruption on FFA) may suggest that overt breakdown of the blood retinal barrier is not prominent in the early-to-moderate stages of CSVD represented in our cohort. Furthermore, the non-significant findings for V2-V1 and arteriovenous nicking indicate that these parameters may lack sufficient specificity for early retinal microcirculatory abnormalities linked to cerebral small vessel damage. Addressing these negative findings provides a more balanced and comprehensive interpretation of the relationship between retinal FFA biomarkers and CSVD burden. The correlation analysis revealed that both ARCT and V1-ARCT were significantly associated with CSVD severity in unadjusted analyses. A key and novel finding was the divergent behavior of these two parameters following multivariable adjustment. The association of ARCT with CSVD burden was abolished after adjustment, indicating that ARCT predominantly reflects systemic circulatory status (including cardiac output and large‑artery stiffness) that is heavily influenced by conventional cardiovascular risk factors. In contrast, V1-ARCT remained independently and robustly associated with CSVD burden, signifying its specificity for localized microcirculatory dysfunction along the retina–brain axis. This distinction highlights that V1-ARCT directly reflects microvascular regulation and endothelial integrity in the small vessel compartments specifically affected by CSVD, representing a core finding that differentiates systemic hemodynamic effects from local microvascular impairment in CSVD. This mechanistic interpretation is supported by early methodological work demonstrating that arteriovenous passage time, the physiological counterpart of V1-ARCT, can be reliably quantified from video-fluorescein angiography and reflects key characteristics of retinal microvascular blood flow, perfusion efficiency, and microcirculatory function [35]. Prolonged V1-ARCT in our study thus indicates impaired microvascular perfusion and regulation within the retina–brain axis, consistent with the microvascular dysfunction underlying CSVD.

The results of the present study demonstrated that increased CRVE and prolonged V1‑ARCT were positively correlated with a higher total CSVD burden score and could serve as positive biomarkers. In contrast, narrowing of the CRAE was negatively correlated with CSVD burden, representing a negative biomarker.The AVR was not included in the final regression model due to high multicollinearity with CRAE and CRVE (VIF > 10). Although AVR is a commonly used composite index in cerebrovascular research, our study indicated that CRAE and CRVE each showed a stronger independent association with CSVD severity.The significant correlation between AVR and CSVD burden in univariate analysis was mainly driven by opposing pathological changes in its two components: decreased CRAE reflecting progressive arteriolar constriction, and increased CRVE reflecting compensatory venular dilation. These findings suggest that arteriolar constriction and venular dilation are simultaneous and interrelated pathological processes in CSVD‑related microvascular dysfunction.Mechanistically, CRAE reflects retinal arteriolar remodeling under chronic hypoperfusion, whereas CRVE indicates venular dilation associated with cerebral microvascular dysfunction. As a relative ratio, AVR may mask the distinct pathological information of arterioles and venules. Therefore, separate assessment of CRAE and CRVE is more pathophysiologically meaningful and sensitive for evaluating CSVD severity.

Our study has some limitations that should be considered. First, this was a single-center retrospective study with a relatively modest sample size, which may reduce statistical power, increase the risk of false-negative results, and weaken the robustness of between-group comparisons. In addition, the single-center design and limited sample size restrict the external generalizability of our findings. Therefore, large-scale, multi-center, and longitudinal studies are warranted to validate these preliminary results in the future. Second, the present study enrolled participants aged ≥ 40 years. Although we strictly included only patients with arteriolosclerotic CSVD and excluded hereditary CSVD, cerebral amyloid angiopathy, migraine-related white matter hyperintensities, and other secondary etiologies, atypical cases may still exist among individuals younger than 50 years, which limits the generalizability of our findings. Our results may therefore not apply to other CSVD subtypes. Future studies may adopt stricter age stratification or enroll only middle-aged and elderly populations for validation, and should investigate subtype-specific retinal biomarker profiles.Third, We did not include potential confounding factors such as BMI and statin use in the model, which may have a certain impact on the stability of the results. Finally, FFA is an invasive procedure, the large scale application for early CSVD screening remains challenging, thus, it may be more suitable for targeted screening in high risk populations.

## Conclusion

In conclusion, the specific retinal microvascular parameters CRAE, CRVE and V1-ARCT are independently associated with CSVD severity in our cross-sectional observational study. These findings support the use of retinal assessment as a potential window to cerebral small vessel health, rather than indicating definitive causal relationships. Future longitudinal and prospective studies are warranted to validate these biomarkers in diverse populations, assess their prognostic value, and clarify the temporal sequence of retinal changes preceding CSVD progression. Incorporating retinal evaluation into clinical practice may improve early CSVD detection and prevention strategies.

## Data Availability

The corresponding author takes full responsibility for and has complete ownership of all data presented in this manuscript. De-identified raw data supporting the findings of this study are available from the corresponding author upon reasonable request to qualified researchers.
